# “Nobody does checkups back there”: A qualitative study of refugees’ healthcare needs in the United States from stakeholders’ perspectives

**DOI:** 10.1371/journal.pone.0303907

**Published:** 2024-06-04

**Authors:** Sarah Yeo, Hannah L. N. Stewart, Ragha Mohan, Kalpana Poudel-Tandukar, Sommer Aldulaimi, Brittany DiVito, Halimatou Alaofè

**Affiliations:** 1 The University of Arizona Cancer Center, Tucson, Arizona, United States of America; 2 Department of Health Promotion and Behavioral Sciences, The University of Texas Health Science Center at Houston School of Public Health, Houston, Texas, United States of America; 3 Department of Public Health Sciences, West Chester University of Pennsylvania, West Chester, Pennsylvania, United States of America; 4 Elaine Marieb College of Nursing, University of Massachusetts Amherst, Amherst, Massachusetts, United States of America; 5 Department of Family and Community Medicine, University of Arizona, Tucson, Arizona, United States of America; 6 Edson College of Nursing and Health Innovation, Arizona State University, Phoenix, Arizona, United States of America; 7 Department of Health Promotion Sciences, Mel and Enid Zuckerman College of Public Health, University of Arizona, Tucson, Arizona, United States of America; Jashore University of Science and Technology (JUST), BANGLADESH

## Abstract

The number of refugees globally grew to 35.3 million in 2022, and many refugees are exposed to various health risks along their migration journey. As a result, they may arrive in host communities with numerous health issues, including communicable diseases and chronic and mental health conditions. Navigating the healthcare system in a host country proves to be a significant challenge for them, leading to delayed care. This qualitative study explored the convolute healthcare needs of refugees in the United States by soliciting insights from stakeholders involved in refugee resettlement and healthcare. In-depth interviews were conducted with fifteen stakeholders who work closely with refugees, including healthcare providers, cultural/clinical health navigators supporting refugees, staff from refugee resettlement agencies and governmental entities, and researchers studying refugee health. Following informed consent, interviews were audio-recorded, transcribed verbatim, and imported into MAXQDA 2022 (VERBI Software) for thematic analysis. The results revealed key themes, including the heterogeneity of refugee populations, limited awareness of preventive healthcare, high prevalence and suboptimal management of chronic conditions, complexity of the healthcare system, lack of follow-up, and language barriers. Further research is warranted concerning the long-term health of refugee populations in the United States. Additionally, more tailored programs involving peer educators are recommended to support refugee communities in navigating the complex healthcare system in the host country.

## Introduction

Globally, the number of refugees experienced an unprecedented surge, reaching 35.3 million in 2022, compared to 26.6 million at the end of 2020 [1]. Many refugees are exposed to various health risks before, during, and after their migration journey [2]. Refugees face significant health challenges, including deprivation during migration, poor living conditions, and inadequate hygiene, increasing susceptibility to infectious diseases [2, 3]. Refugees with pre-existing conditions, such as cardiovascular disease and diabetes, confront exacerbated challenges due to disrupted treatment and management during the arduous journey. They could also come from countries that lack established healthcare systems for regular screenings of chronic diseases and conditions [3]. Pregnant women fleeing conflict may struggle to access maternal care. Long-term exposure to violence and traumatic events also contributes to psychological distress, including post-traumatic stress disorder, depression, and anxiety [2, 4]. As a result, refugees arrive in host communities often with a myriad of health issues, including communicable diseases and chronic and mental health conditions [3, 5, 6].

The United States (US) has resettled over 3.2 million refugees since the enactment of the Refugee Act in 1980. Various entities and stakeholders in the country are engaged in the processes of refugee resettlement and guiding refugees through the healthcare system. As of February 2023, there are ten refugee resettlement agencies in the country [7], each offering diverse services to facilitate resettlement. These services include cultural orientation, counseling, employment assistance, housing support, and aiding in access to health services for newly resettled refugees [8]. Some clinics go a step further by providing specialized services tailored to refugees, offering on-site interpretation services and assistance with healthcare navigation. Additionally, the Office of Refugee Resettlement within the US Department of Health and Human Services collaborates closely with both refugee resettlement agencies and state/local governments to facilitate the placement and resettlement of refugees in the country [9].

Despite these efforts, challenges persist for refugees resettled in the US. While refugees receive time-limited health insurance upon arrival [10], the insufficient funding dedicated to refugees and the complexity of the US health system mount innumerable barriers to accessing healthcare [11]. Many refugees are unaccustomed to the US health delivery model characterized by preventive care, referrals to outside specialists, and multiple follow-up appointments [12]. Consequently, refugees often struggle to navigate the healthcare system and delay seeking care [13]. A study examining insurance coverage among refugees in the US discovered that 49 percent of them lacked insurance [14]. Furthermore, the inadequate cultural competence among health care providers and language barriers create additional challenges for refugees [15].

The majority of research conducted on the healthcare needs of refugee populations in the US has primarily elicited perspectives of refugees. Despite their valuable lived experiences, refugees may lack awareness of the available resources in the US, may not fully comprehend the potential structural factors influencing their healthcare access, and might not be able to compare their situation with other cultures or ethnic groups. Stakeholders possess distinctive and valuable insights into the challenges faced by refugees in the US, both at the individual and systemic levels, derived from active engagement with refugee communities and contributions to the resettlement process at various stages [16]. This study aimed to explore their unique perspectives on the healthcare needs of US-based refugees and enhance the readiness of the healthcare system to address these needs. This paper focuses solely on refugees and does not include other immigrant groups such as asylum-seekers or undocumented workers due to their distinct characteristics and varying entitlements to services [15]. In this study, the definition of refugees aligns with the one outlined in the US Immigration and Nationality Act, which describes a refugee as someone who:

- Is located outside of the United States
- Is of special humanitarian concern to the United States
- Demonstrates that they were persecuted or fear persecution due to race, religion, nationality, political opinion, or membership in a particular social group
- Is not firmly resettled in another country
- Is admissible to the United States.

Additionally, in this study, a "stakeholder" is defined as an individual or a group with a vested interest in refugee health, actively engaging and interacting with refugee populations, and/or contributing to the resettlement process at different stages.

## Methods

### Study participants

Study participants were purposefully selected based on their occupational roles to elicit different perspectives related to refugee healthcare needs and navigation. The predefined categories included health care providers, cultural/clinical health navigators working with refugees, staff from refugee resettlement agencies and governmental agencies, and researchers studying refugee health. The inclusion criteria were those with more than three years of experience working with refugees in the US. Recruitment was conducted in multiple ways, including community partners sharing contacts with potential participants and using snowball sampling strategies. Speakers at academic conferences and webinars about refugee health were also contacted to participate. The sample size for this study was determined by the existing literature [17], and fifteen stakeholders were recruited and consented to be part of the research.

### Data collection

Data were collected through in-depth interviews with a semi-structured interview guide. The interview guide included demographic questions, followed by questions on their roles and responsibilities and the perceived needs and challenges of refugees that they worked with. More specifically, the following themes were explored.

- Roles and responsibilities of the study participants
- Experiences working with refugee populations
- Important issues, unmet needs, and challenges concerning refugee health
- Existing resources and services aiding refugees in accessing healthcare
- Recommendations for enhancing healthcare access and utilization among refugee populations

All interviews were conducted by the first author over Zoom from June 2022 to March 2023. Prior to the interview, the first author went through the consent form, and upon the recorded verbal consent, each interview began. The interviews lasted approximately 30–80 minutes and were conducted in English, audio-recorded, and transcribed.

### Data analysis

The transcripts were imported into MAXQDA 2022 (VERBI Software) for thematic analysis [18]. The authors (SY, HS, and RM) collectively reviewed transcripts, generating codes based on existing literature [18–20]. The derived coding framework was applied to the initial transcript after a consensus was reached on the codes to be included. Subsequently, the authors convened to evaluate the necessity of additional codes, refining and amending the codebook. This updated codebook was employed for coding the second and third transcripts, followed by iterative refining processes. Any newly identified themes or codes were discussed among the authors and incorporated into the codebook. After the codebook was finalized, the first author used it to code the remaining set of manuscripts. The manuscript was shared with the study participants for member checking and to ensure the trustworthiness of the results. The quotations from the participants were used verbatim, even if they included grammatical errors. In instances where ascertaining the context proved challenging, the authors included additional words within parentheses. Ethical approval for the study was granted by the Institutional Review Board (IRB 2104716241). The reporting adhered to the Standards for reporting qualitative research (SRQR) checklist whenever relevant [21].

## Results

### Participant characteristics

Fifteen stakeholders participated in this study: four individuals serving as cultural/clinical health navigators, three researchers, two representing refugee resettlement agencies, two from a state governmental body responsible for refugee affairs, and four health care providers. The average age of the participants was 43.8 years, with 12.3 years of experience ranging from 2 to 40 years (Table 1). Although the eligibility criteria required a minimum of three years’ experience, one participant with two years’ experience was included due to her valuable lived experiences as a refugee. All cultural health navigators were refugees or immigrants to the US, and one health care provider was a refugee from Afghanistan.

**Table 1:** The characteristics of study participants (n = 15).

	n
Age (mean, range)	43.8 (27–70)
Gender	
Female	13
Male	2
Occupation	
Health care provider	4
Cultural/clinical health navigator	4
Researcher	3
Refugee resettlement agency staff	2
Governmental officer	2
State	
Arizona	10
California	2
Massachusetts	1
Maryland	1
Pennsylvania	1
Years of experience (mean, range)	12.3 (2–40)

The subsequent sections delve into the prevalent themes such as the heterogeneity within refugee populations, limited awareness of preventative healthcare, the high prevalence and suboptimal management of chronic illnesses and conditions, complex healthcare systems and a lack of guidance to navigate them, and language barriers. Furthermore, a noteworthy approach endorsed by various stakeholders is also examined.

#### Heterogeneity among refugee populations

Many participants discussed the ways in which heterogeneity among refugee groups impacted their healthcare needs and access to care. The characteristics and healthcare needs of refugees varied significantly based on factors such as educational levels, socioeconomic status prior to resettlement, geographical setting in their home countries (urban or rural), English proficiency, or the migration history of their ethnic group. For example, some refugees may have differing perspectives on medical procedures such as C-sections, depending on their geographic origin, even if they are from the same country. One health care provider who migrated from Afghanistan as a refugee highlighted this issue.

It depends on where you live. In urban areas, C-section is accepted, and women feel like it’s an easier way to get delivery. But in a remote area, it’s very difficult to formulate a C-section as an option… the reason is (that) women work a lot in a farm. And they (men) don’t want women to be long time in the bed. (Men are afraid that) Her body now is weak so she cannot work as strong as before. So they are afraid of it (C-section). (Interviewee 4, Health care provider)

Moreover, the extent of social support that refugees have access to in the host country appeared to differ based on the history of migration and resettlement modalities—whether they arrive as families or individuals, or in a significant concentration within the same geographical area. For instance, when a substantial number of ethnic groups relocate and form an ethnic enclave, they tend to provide mutual assistance in navigating the system. This support can be very helpful for the refugees if it exists in the area where they are resettled. One researcher explained how this has shaped the Bhutanese refugee community that she worked with.

They are with all their families and community members here. That’s why the family members are available to help … in the case of Bhutanese community, at least they have some support systems within the community to take (medical) appointments and even they try their best to help relatives or somebody for the transportation. (Interviewee 3, Researcher)

#### Unfamiliarity with preventive care

Numerous participants highlighted that refugees are not familiar with preventive care. Many refugees come from “areas where the healthcare system broke down” so they “don’t have a functioning primary care and preventive healthcare system (Interviewee 7, Researcher).” Thus, some people were often dependent on traditional medicine when they were ill, as noted below.

Because of the lack of healthcare system back home, they don’t do checkups. So, women don’t do mammograms at all. They never even heard of mammograms. And even all other diseases they don’t check. All they use is traditional medication, something to help them when they’re really, sick. But nobody does checkups back there. (Interviewee 12, Clinical health navigator)

Another stakeholder explained how unfamiliarity with preventive care causes challenges. Refugees may be confused when their doctor recommends screenings, labs, or other preventive care options they are not accustomed to. A refugee settlement agency staff member said, *“*I would say in a lot of cultures, that (preventive care) is not something they’ve been exposed to, and they don’t really understand why they would need to do that.” (Interviewee 13, Refugee resettlement agency staff).

#### Chronic conditions and diseases

Many stakeholders noted that refugees are experiencing “a lot of chronic health conditions,” such as cardiovascular disease, diabetes, and asthma. A family medicine doctor with over 15 years of experience treating and screening refugees made the following observation.

I would say, it’s not just infectious disease that we see, we see a lot of chronic health conditions. Whether that’s cardiovascular disease, or smoking, or obesity, or diabetes, or asthma, there’s a lot of chronic health conditions that we see in the refugee community. (Interviewee 2, Health care provider)

Lifestyle changes after resettlement were identified as a contributing factor to chronic diseases such as diabetes. One researcher and health care provider shared their observations on this issue.

Almost every house has one person with diabetes now because of lots of sweets, cake, and junk. After coming here, they told me that they’re eating more processed food and that they’re eating that type of (junk) food also. (Interviewee 3, Researcher)

A lot of times patients don’t have diabetes because they have not been eating junk and soda and whatever (in their home country). And then they come to the US and they develop it here because all of a sudden there’s availability of all of this kind of stuff. (Interviewee 6, Health care provider)

However, these health conditions often remained inadequately managed due to patients’ unfamiliarity with the US healthcare system and limited knowledge of the conditions. A clinician described how their lack of knowledge about the US pharmacy system makes it challenging to manage their chronic health condition. One respondent stated, “they finished the bottle (of medicine to manage a chronic condition) they got the last time, and they didn’t know how to get the next month’s supply" (Interviewee 1, Health care provider). In particular, acymptomatic chronic conditions or diseases posed significant obstacles to treatment adherence.

The main challenge is getting the client to understand just because you feel okay, doesn’t mean you don’t have hypertension. It’s hard … They just don’t see any reason why they should take medication. (Interviewee 13, Refugee resettlement agency staff)

#### Refugees lost in a health surveillance system and follow-up

Despite stakeholders anecdotally reporting a high prevalence of chronic conditions and diseases, the long-term health outcomes of refugees remain largely unexplored. This is partly due to inadequate classification that fail to capture refugee status and the absence of follow-up procedures. A faculty member, who has been studying immigrant and refugee health for over 40 years, expressed her concern regarding this issue.

One issue is that whether a patient is a refugee or not is not very well captured in our electronic health system. So you sometimes may know where a person is from, you may know the primary language, but that doesn’t tell you whether it is a diplomat from Iraq or whether it is a refugee. So it makes it very difficult to identify gaps and to monitor (issues) in refugees over time. So if somebody is a Somali refugee, once they are in the system, they are just identified as black… That is a big gap that needs to be addressed. (Interviewee 7, Researcher)

When asked about the existence of any monitoring or surveillance mechanisms to assess the long-term health of refugees, including chronic disease prevalence and risk factors, both a governmental officer responsible for refugee resettlement programs and a staff member from a refugee resettlement agency confirmed that no such system is currently in place.

No, there’s no following that. (Interviewee 9, Governmental officer)

No, nobody from our agency is following them. (Interviewee 13, Refugee resettlement agency staff)

#### Complex healthcare system and lack of orientation

Participants perceived that refugees were struggling to navigate the complex healthcare system in the US. Health care providers also acknowledged that the health system poses challenges even for well-informed medical professionals. One provider stated, “our healthcare system is very complex, and most Americans barely understand it. I’m a well-educated American with a lot of health degrees, and I feel like I barely have my head wrapped around our healthcare system” (Interviewee 1, Health care provider).

For refugees who have recently arrived in the US, this complexity is even more overwhelming. Many refugees originated from countries where scheduling medical appointments was unnecessary, and hospitals served as central locations to see patients, obtain laboratory tests, and acquire prescribed medications. However, in the US, refugees have to find a primary care provider, schedule appointments, pick up medicines or undergo laboratory tests from different locations, and handle complicated tasks such as medicine refills, referrals for specialty care, or addressing medical billing errors. One clinician highlighted how this can create challenges for a patient who is not familiar with different levels of healthcare.

Our healthcare system is extremely difficult to navigate for anybody, including me who’s a doctor. It’s very hard to navigate our system. It’s a mess. … And so when you say like, "Oh, I’m going to refer you to a specialist on this thing," they’re like, "But you’re a doctor, can’t you just take care of that thing for me?" … because they just are not used to having the number of different kinds of doctors and services that we have. (Interviewee 6, Health care provider)

Despite these complexities, many participants voiced concern about the insufficient and brief cultural orientation provided to refugees upon arrival to the US. A resettlement agency staff member stated that the health orientation provided to refugees is sometimes “an hour and that’s it” (Interviewee 8, Refugee resettlement agency staff). The brevity of the orientation is further complicated by the timing of its delivery, which is expected to occur within 30 days after arrival for the majority of refugees. Since this orientation is provided at a period when refugees are grappling with acquiring fundamental knowledge about living in the US, they may find it difficult to remember the information from their health orientation as voiced below.

All refugees are supposed to get a health orientation within 30 days of arrival, but I feel like there is so much happening in those first 30 days that I don’t know how they possibly would retain any of that. (Interviewee 1, Health care provider)

As a result, refugees, often with limited English proficiency, may struggle to recall the information from their health orientation when they need it.

For an English speaker, it’s easy to remember what urgent care is. But for a Persian or a Pashto speaker, oh my God, it’s very difficult to remember what urgent care is and what primary care is. And then they will mix up what was the Medicaid or Medicare…It’s very difficult. (Interviewee 4, Health care provider)

#### Language barriers

While many study participants emphasized the importance of language skills for navigating life and healthcare in the US, even refugees who become long-term residents continue to encounter language barriers. Study participants noted that, despite the availability of English classes for refugees, many encountered challenges such as childcare, transportation, and conflicts with work schedules, which hindered their participation. Online options were only accessible to individuals who had both a computer and internet access and were comfortable using them. A stakeholder explained:

Their classes are during the day, they sometimes don’t provide childcare, and they don’t provide transportation. So, the refugees have to be able to take the bus. Many times, in my experience, the refugees would be very good about going initially, but then if they get a job, they really just don’t have the time to go. (Interviewee 13, Refugee resettlement agency staff)

This inability to engage in language classes can significantly impact refugees’ lives. Some participants mentioned that limited language skills hinder some refugees from obtaining US citizenship, thereby denying them access to benefits available for citizens. Learning a new language can be particularly challenging for older individuals or those who struggle with literacy in their native language.

If you are in your forties or fifties, you never go to school, it’s impossible to learn English within a couple of months while doing manual labor job. And the immigration cannot accommodate them to get citizenship unless they pass the test. So, some people stay here for 10, 20 years without getting citizenship because they can’t learn English. They can speak it, but they can’t read or write. So how do you ask someone who is 60 to learn English and write English when they don’t even know how to write their own language? (Interviewee 12, Clinical health navigator)

Additionally, while health care providers receiving federal funding are required to offer interpretation services [22], access to the services beyond clinics closely associated with refugees remained challenging, particularly for specialized medical care.

Once you get outside the few clinics that specialize with refugees, it’s extremely difficult. If a client is referred to a specialist, chances are that specialist is going to say, if you said, do you provide interpretation? They’ll say, "Yeah, we speak Spanish. Great. Well, we don’t need Spanish." It is a huge challenge. (Interviewee 13, Refugee resettlement agency staff)

#### Promising practices such as cultural health navigators, peer educators, and community health workers

While numerous participants identified various struggles encountered by refugee communities, many also highlighted promising practices that could potentially contribute to serving these communities. Some clinics employed cultural navigators proficient in various languages to help refugees navigate the system. They assisted refugees in setting up appointments, organizing transportation, accompanying them to medical visits and laboratory tests, and even aiding them in enrolling in social programs. For many refugees, having someone to help them navigate their healthcare needs makes them “more comfortable” (Interviewee 14, Cultural navigator).

Another program connected newly arrived refugee women with other refugee women who had been in the country for longer to provide support in their native language and share insights from their personal experiences. A researcher described, “we called it mentor sister program so that refugee women who have been here for a longer time get trained to work with new arrivals as well” (Interviewee 7, Researcher).

## Discussion

This study identified the needs and challenges that refugee communities often face, perceived by the stakeholders that interact with refugees on a regular basis. Refugees are often faced with a significant level of difficulty navigating the healthcare system in the country, which is vastly different from their country of origin. Nevertheless, the current cultural orientation and training are insufficient to address their unique needs, and language barriers often exacerbate the challenges accessing and utilizing healthcare services. Additionally, their knowledge of preventive healthcare and chronic diseases tends to be limited and a follow-up is lacking, despite increasing needs resulting from changes in their norms and practices.

The results resonate with previous studies. Refugees were more likely to report chronic conditions than any other groups of immigrants in the US, and refugees with chronic conditions were more likely to lack health insurance, making them more prone to disease burden [14]. Despite this increased risk, they were less likely to use preventive health services due to limited knowledge and unfamiliarity with the regular screening practices [23]. Language barriers further impede effective communication between health care providers and patients, leading to suboptimal care and dissatisfaction with care and poor chronic disease management, delaying access to care, and limited use of preventive care [23, 24]. A systematic review, which compiled studies addressing healthcare service challenges for immigrants from the perspective of healthcare providers, also highlighted language and cultural barriers, as well as unfamiliarity with the host country’s health system, as common obstacles [15]. Nonetheless, despite these challenges, several studies identified potential facilitators that could enhance immigrant access to and utilization of healthcare services. These include clinician training in culturally competent care, better access to language interpretation services, and orientation to the healthcare system [25]. Additionally, favorable macro-level factors such as the political climate, government policies regarding immigrants, and ethnic networks within the host country could also positively impact healthcare service utilization among immigrants [26]. A future study could benefit from exploring these potential factors in enhancing healthcare access and utilization among refugees. Furthermore, given refugees’ lack of familiarity with preventive care, the high incidence of chronic conditions and diseases reported by the stakeholders, and the changes in their lifestyles and dietary patterns, further research is warranted to understand their long-term health status, specifically regarding the prevalence and risk factors of chronic diseases.

This study also highlights the importance of a prolonged series of trainings for refugees to help them navigate the complex healthcare system in the country. Additionally, more sensitization and education concerning preventive healthcare and chronic diseases need to be in place. More importantly, these programs need to be tailored to their unique circumstances and varying levels of competency and literacy.

One promising approach could be supporting refugees through cultural health navigators or peer educators, as recommended by various stakeholders. Nevertheless, its potential remains insufficiently explored and assessed. A scoping review that examined different interventions aimed at enhancing immigrant health highlighted that only 8 percent of these interventions were related to peer navigation [27]. Furthermore, even among studies that employed this approach, there were notable methodological limitations such as lack of comparison groups, small sample sizes, and reliance on self-reported outcomes, which could introduce bias [27]. Therefore, it may be beneficial to validate its potential through a more rigorous study design as compared to other models of care.

This study stands out as one of the few studies that incorporated perspectives from diverse stakeholders and refugees themselves within the context of refugee health in the US. However, due to the qualitative nature of the research, the findings may not be applicable to other populations. Nevertheless, the refugee communities served by the stakeholders encompassed a broad spectrum, including refugees from Africa, Southeast Asia, and the Middle East. Furthermore, the reported high prevalence of chronic diseases and conditions is anecdotal and may be biased due to the characteristics of individuals seeking care compared to those who do not.

## Conclusions

This study underscores the importance of addressing the unique needs and challenges faced by refugee communities in the US. These challenges include a lack of awareness regarding preventive healthcare, a notable prevalence of chronic conditions with suboptimal management, lack of follow-up, the complex healthcare system, and language barriers especially when seeking specialist care. In addition to their unfamiliarity with the healthcare system, they are grappling with new challenges posed by changes in their lifestyles. Additionally, despite the substantial number of refugees resettled in the US, there is a lack of systematic understanding of the long-term health of these newcomers. There is a pressing need for more comprehensive efforts to meet their unique needs, as well as capture their long-term health outcomes, particularly for chronic diseases and conditions. To address various challenges, programs targeting refugee populations must recognize the heterogeneous nature of these groups and attend to diverse needs, even when they are from the same country. Furthermore, it would be beneficial to explore and assess the effectiveness of the approach utilizing cultural health navigators or peer educators. Finally, it is essential to provide tailored support that considers varying levels of competence and circumstances, extending beyond the initial resettlement years, to ensure that refugees are leading healthier lives in their new communities.
